# Organic Foreign Body in the Eye, a Diagnostic Challenge: A Case of a Wooden, Intra-orbital Foreign Body Presenting at the Emergency Department

**DOI:** 10.7759/cureus.75011

**Published:** 2024-12-03

**Authors:** Aaruran Nadarajasundaram

**Affiliations:** 1 Emergency Department, St Thomas' Hospital, London, GBR

**Keywords:** imaging modalities, infection, intra-orbital foreign body, multidisciplinary care, orbit, organic foreign body, penetrating eye injury, trauma

## Abstract

Intra-orbital organic foreign body injuries occur within the eye but without the involvement of the orbit itself.

A 39-year-old man self-presented to the emergency department complaining of sudden onset of pain surrounding his left eye and of reduced vision. The initial examination was unremarkable except for two healing lesion marks above his left upper eyelid. Some swelling with erythematous skin changes was also noted. Computer tomography did not identify a conclusive cause. The patient was assessed by an Ophthalmologist in Eye Casualty and commenced intravenous antibiotics for infection secondary to an intra-orbital wooden foreign body. The patient underwent surgery to remove the foreign body, experienced no postoperative complications, and was discharged following a brief medical admission for intravenous antibiotic administration, with vision returning to normal in the affected eye.

This case report showcases the difficulty of such cases, given the organic nature of the foreign body. It also highlights the need for high clinical suspicion with thorough history-taking, as well as physician collaboration, to ensure organic foreign bodies are considered in similar presentations or cases.

## Introduction

Intra-orbital foreign bodies are defined as foreign bodies located within the eye but without involvement of the orbit itself [1]. Foreign bodies can be categorized based on their composition as 1) organic, such as wood and plant matter, 2) inorganic, such as glass and plastic, or 3) metallic. Imaging is a key component in the investigation of suspected foreign bodies in the eye. However, for cases in which the foreign body has an organic composition, imaging is more difficult due to the low density of the foreign body, which is harder to visualize with certain imaging modalities. This results in delays in appropriately diagnosing and managing the issue. Organic foreign bodies left in situ can lead to inflammatory responses and complications for the patient, such as infection and abscess formation, highlighting the need for urgent identification and surgical removal [2].

The case below illustrates the challenges in such cases and emphasizes the importance of patient work-up and multidisciplinary collaboration in cases involving suspected organic foreign bodies in the eye.

## Case presentation

A 39-year-old man self-presented to the emergency department in the early morning, complaining of sudden-onset pain surrounding his left eye as well as a change in vision. He reported no significant medical history and had no previous surgical or ocular interventions. He explained that he had been working in his garden the previous day, which involved pruning and handling thorny plants as well as cutting small branches from one of his trees. He explained that he had felt something hit his left eye toward the end but noticed no symptoms or change in his vision; hence, he did not seek medical attention at the time.

On inspection, swelling was noted around his left upper eyelid with some erythematous skin changes, as well as two lesions with some scab formation over them. On closer examination, there were no remnants or evidence of any retained foreign body at the site. The remainder of the eye examination proved to be unremarkable, with no cranial nerve or visual field defects noted. However, he had persistent pain around the eye with tenderness on soft palpation; as a result, deep palpation was not possible. Additionally, he had reduced visual acuity in the affected eye, measured at 20/30 OS as compared to 20/20 OD.

**Figure 1:**
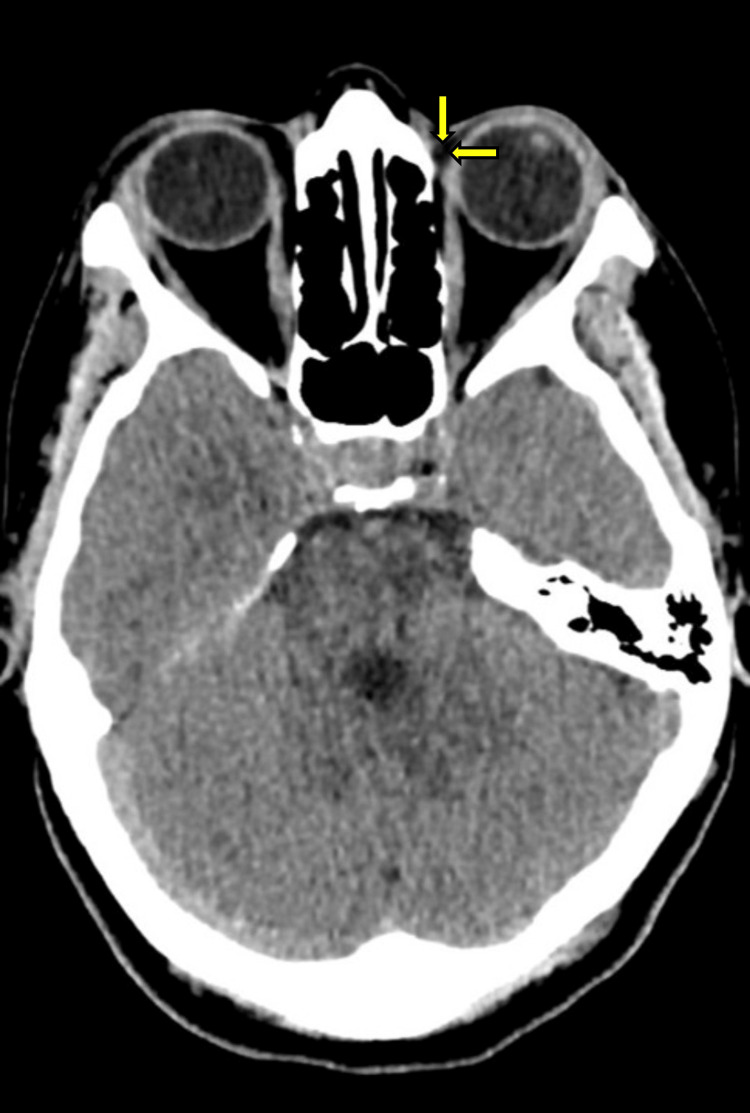
Computer tomography scan cross-section of the patient's head. Yellow arrows indicate the eventual location of the wooden foreign body that was not detected in this scan.

The patient underwent a computer tomography (CT) to rule out foreign bodies, which was unremarkable and found no definitive foreign body. The arrows in Figure 1 point to the unremarkable area on imaging, where the organic foreign body was subsequently located. Given the ongoing visual symptoms and lack of a definitive cause noted on imaging, an on-call ophthalmologist was contacted in the morning. The advice was for the patient to be reviewed in Eye Casualty in view of the current findings. On repeat examination in Eye Casualty, a small firm mass was noted on deeper palpation of the swelling above the left upper eyelid. Given the patient’s activities prior to symptoms, the patient was offered a prophylactic tetanus vaccine and taken to theatre for surgical removal of the retained foreign body. This revealed a small, wooden intra-orbital foreign body (1cm in length with a diameter of 1mm) that had penetrated and embedded itself around the upper eyelid, and which was successfully removed. The patient was subsequently discussed and admitted under the medical team for intravenous antibiotics before being discharged. No further complications were noted, and vision in the left eye returned to his usual baseline of 20/20.

## Discussion

Globally, ocular injuries remain a leading cause of vision loss and often require surgical intervention with extensive subsequent rehabilitation and associated socioeconomic costs [3]. Furthermore, the Global Burden of Diseases, Injuries, and Risk Factors Study in 2019 found an increased number of eye injury cases, with foreign bodies categorized as one of several main causes [4]. As mentioned above, surgical removal is recommended as a treatment for wooden foreign bodies, owing to the increased risk of infection and associated tissue necrosis due to their organic composition [5,6]. This case highlights the challenges of cases involving organic foreign bodies. It highlights the difficulty of identification and appropriate treatment initiation, as well as the importance of thorough history-taking and clinical examination, as these foreign bodies can be missed in certain imaging investigations.

Given the non-specific range of clinical presentations in such cases, meticulous history-taking and clinical examination are vital, especially in cases in which there is evidence of penetrating orbital or lid injuries. A review of the literature shows that the extent and severity of penetrating injuries to the orbit are underestimated, which further necessitates the emphasis on early diagnosis and treatment [7,8]. In this case, the reduced visual acuity may be in keeping with the traumatic event and associated oedema, with visual acuity improving post-surgical intervention and antibiotic treatment.

A key learning aspect of this case is the importance of a multidisciplinary team working with a patient attending a general emergency department instead of an out-of-hours eye casualty service. The experience of emergency medicine (EM) physicians can vary from relatively newly qualified doctors to specialty trainees and consultants: a recent systematic review in the UK noted low confidence among EM physicians in managing ophthalmic emergencies [9]. This, coupled with the relative difficulty of early detection of retained organic foreign bodies, highlights the importance of close collaboration and further training among ophthalmologists and EM physicians to ensure that timely management is commenced in such cases. The collaborative work between EM physicians and ophthalmologists in this case allowed for swift initial work-up and specialist review; the patient received treatment promptly, and the visual acuity in the affected eye returned to normal over the course of treatment.

Secondly, the case stresses the importance of not relying solely on radiological imaging in the diagnosis of foreign body injuries. While the role of imaging should not be understated, a review of cases and literature has found examples of other ocular-retained foreign body cases in which there were no initial conclusive findings on scans, but in which patients subsequently re-presented with further complications that prompted repeat imaging and interventions [10-13].

Plain X-ray images and CT imaging are common first-line imaging modality choices for patients presenting with suspected or clinically visible foreign body injuries [10]. Overall, non-contrast CT brain and orbit imaging of the coronal, axial, and parasagittal views remains the gold standard in imaging modality [14]. There have been arguments questioning the role of plain X-ray imaging in foreign body cases, given that subsequent CT imaging would be needed for dimensional localization in advance of anticipated surgical intervention and for detecting undetected foreign bodies missed in previous X-rays. Conversely, it is important to acknowledge that this would likely cause prolonged time intervals between presentation and treatment initiation while CT images are being reviewed [10]. Importantly, as seen in this case, in some instances unremarkable CT imaging does not rule out the presence of retained foreign bodies. Organic foreign bodies pose this difficulty due to their low density, which gives an appearance similar to adipose and air, allowing the object to blend in with the surrounding orbital structures and remain undetected in the early stages [15].

Other imaging modalities to be considered include ultrasounds and magnetic resonance imaging (MRI). It is important to note that the results of ultrasound imaging are heavily operator-dependent and unreliable, and ultrasound examination poses the risk of potentially moving or dislodging any suspected foreign bodies. On the contrary, MRI is a more suitable imaging modality for detecting organic foreign bodies, as they appear hypodense in comparison to the surrounding intra-orbital tissues [2]. It is important to ensure that any foreign body in question is neither metallic nor ferromagnetic, as these are contraindications for MRI scanning. As a result, MRI could be utilized in cases in which organic foreign bodies (particularly wood) are suspected following unremarkable CT findings to ensure that such foreign bodies have not been missed [16].

## Conclusions

This case summarizes the challenges faced when encountering intra-orbital organic foreign bodies and provides several learning opportunities. The management of the issue relies on early diagnosis and intervention. As this case shows, while imaging plays a significant role in the diagnostic process, there should not be an over-reliance on imaging alone. Meticulous history-taking, physical examination, high clinical suspicion, and appropriate imaging modalities all play key roles in the work-up of such cases. This case also emphasizes the importance of close collaboration among different specialties, as well as the need for continued education and training to ensure the continued professional development of physicians and the provision of high-standard care for patients.
